# Supplementary material for: Investigating Chaperone like Activity of Green Silver Nanoparticles: Possible Implications in Drug Development
Source: Molecules. 2022 Jan 29;27(3):944. doi: 10.3390/molecules27030944 (PMC8838336; doi:10.3390/molecules27030944)
Supplement: Supplementary file 1 [file molecules-27-00944-s001.zip › molecules-1558138-supplementary.pdf]

SUPPLEMENTARY FILE

Investigating Chaperone like Activity of Green Silver Nanoparticles: Possible Implications in Drug Development

Mohd Ahmar Rauf[1*], Md Tauqir Alam[2*$], Mohd Ishtikhar[3], Nemat Ali[4], Adel Alghamdi[4], Abdullah F Al Asmari[4$]

[1]Department of Pharmaceutical Sciences, Wayne State University, Detroit, MI, USA
[2]Department of Biochemistry, Aligarh Muslim University, Aligarh, UP, India
[3]Department of Biochemistry and Biophysics, Texas A&M University, College Station, Texas-77843, USA
[4]Department of Pharmacology and Toxicology, College of Pharmacy, King Saud University, P.O, Box 55760, Riyadh 11451, KSA
*Authors contributed equally
$ Corresponding Author

Materials

Human serum albumin (HSA, catalogue no. A1653-1G), ANS, ThT, CR and Silver nitrate (AgNO3, ACS reagent ≥ 99.9%), ANS (molecular formula $C_{16}H_{13}NO_3S$) (Thioflavin T), Congo red were purchased from Sigma Aldrich were purchased from Sigma Aldrich (USA). Therest of the chemicals as urea, sodium phosphate monobasic and dibasic salts were of analytical grade and purchased from Sisco Research Laboratories (SRL, Mumbai).

Biological synthesis of B-AgNPs by *Rosa* (Rose) petals: B-AgNPs were synthesized via reducing the silver precursor, silver nitrate, mediated by *Rosa* petal extract (AgNO3). The synthesis was carried out by incubating a fixed volume of silver nitrate (1mM AgNO3) with various quantities of the OS stock (150–500µL). Water was used to adjust the final volume of the reaction mixture to 5 mL. To achieve B-AgNPs, the reaction mixture was continuously agitated at room temperature for the specified time duration. The sample was centrifuged at 10,000 g for 10 minutes to remove the unwanted plant products/materials. Finally, the pellet was dried at 80°C to obtain the nanoparticles. The synthesized B-AgNPs were then characterized employing TEM and DLS techniques for their shape and size.(Ahmar Rauf et al. 2019; Alam et al. 2018)

Characterization of B-AgNPs

UV-Visible spectroscopy

The UV-VIS absorption spectra of silver ions were examined to determine their biogenic reduction to colloidal nanostructures (B-AgNPs). UV-Visible scanning of the incubation mixture at different time points was used to determine the kinetics of B-AgNPs production (Alam et al., 2018a).

Transmission Electron Microscopy (TEM) and Dynamic Light Scattering (DLS)

The hydrodynamic particle size was studied in Beckman Coulter Delsa Nano DLS particle size analyzer (Miami, FL) at ambient temperatures. The data were evaluated in their default mode of operation. The average of twenty runs (performed in triplicate) was used to determine the size of the as-synthesized B-AgNPs. The morphology of the nanoparticles was analyzed by transmission electron microscopy (TEM, H-7500, Hitachi Ltd, Tokyo, Japan)..(Ahmar Rauf et al. 2019; Oves et al. 2019)

Protein sample preparation

Stock solution (5mg/ml) of HSA was prepared in a sodium phosphate buffer of pH 7.4 and then left overnight for dialysis. Lowry's assay was used for determining the concentration of protein stock solution and the same was cross-verified by using an extinction coefficient of 2.64 ml mg^{-1} cm^{-1} pH 7.4 by UV-VIS 1700 (Shimadzu, Japan). The samples of HSA were prepared with increasing concentrations of B-AgNPs along with the fixed concentration of 5 M urea and were incubated at 37ºC for 96 hours under continuous stir at 40 rpm (Alam et al. 2018).

In vitro exploration of role of B-AgNPs on the aggregation pathway of HSA

Turbidity assay

The preliminary conformational change analysis in the native BSA in the presence of 5M urea and increasing concentrations of B-AgNPs was done by performing turbidity assay. The samples of BSA prepared using 20 mM sodium phosphate at pH 7.4 were incubated at 37ºC for 10 days in the presence of fixed concentration of 5M urea and increasing concentrations of B-AgNPs. The absorbance was taken at 350 nm using UV-Vis-1700 spectrophotometer (Shimadzu, Japan) using proper blank of native BSA in the same buffer (Alam et al. 2018).

Anilino-1-naphthalene-sulphonic acid (ANS) fluorescence measurements

ANS (molecular formula $C_{16}H_{13}NO_3S$) is a dye that binds to the hydrophobic regions of a polypeptide. Spectro fluorophotometer (RF-5301, Shimadzu, Japan) was used for taking ANS fluorescence spectral measurements by setting excitation wavelength at 380 nm and emission at 400-600 nm. The excitation slit width was set at 10 nm while the emission slit width was set at 5 nm (Alam et al. 2018).

Far-UV circular dichroism analysis

Changes in the secondary structure of HSA was analyzed first by Far-UV CD spectral analysis. Spectropolarimeter (JASCO J-815) equipped with a Jasco Peltier type temperature controller (PTC-424S/15) and calibrated with ammonium d-10-camphor sulfonate was used for taking spectra. Measurements were taken at 25˚C and the scanning speed was 100 nm/min with a response time of 1 s. Scanning was done between 190 and 250 nm. An average of four scans were taken for each spectrum to minimize the experimental errors. Results are expressed in terms of MR(Alam et al. 2018).

Transmission electron microscopy

Finally, TEM analysis was done to visualize the native and incubated samples of HSA in the presence of different increasing concentrations of B-AgNPs. 10 µl of the sample was placed on a carbon-coated copper grid and left to absorb for about 1 minute. The grid was then washed with distilled water and air-dried then it was again negatively stained with 2% (w/v) aqueous solution of uranyl acetate for about 45 s. After removing excess stain the grid was air-dried and analyzed using JOEL JEM-2100 (Japan) transmission electron microscope operating at 200 kV(Alam et al. 2018).

Assay for cell viability using MTT assay

The MTT (3-(4,5-dimethylthiazol-2-yl)-2,5-diphenyltetrazolium bromide) assay was used to investigate the toxicity of HSA aggregate on astrocyte cells. In 96-well plates, cells were grown in DMEM-F-12 (10% FBS) medium until they reached 70% confluency. Cells were rinsed with sterile PBS and then treated for 24 hours at 37°C in 5% CO_2 with early-stage HSA oligomers

produced in the presence/absence of B-AgNPs. The detailed protocol has been described in the SI file. The cells were washed to eliminate any remaining HSA oligomers. The cells were then cultured for 24 hours in a new medium. The cells were washed before treatment with MTT solution (5 mg/mL in PBS) for viability testing. The cells were then cultured for an additional 4 hours at 37°C. Following that, the supernatant was thoroughly aspirated. Cells were then solubilized in DMSO. At 570 nm, the complex, purple-coloured formazan produced was measured. As a positive control, healthy cells that had not been exposed to HSA aggregates were employed.

Results

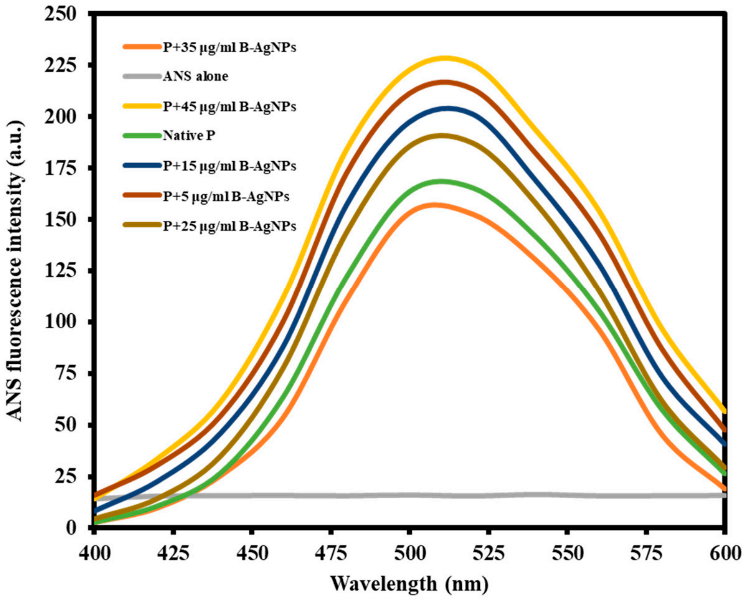

Figure S 1: ANS fluorescence spectra of native HSA, and HSA incubated with increasing concentrations of B-AgNPs and ANS alone. The excitation wavelength was 380 nm, and the emission wavelength was between 400 and 600 nm. The width of the excitation slit was set to 10 nm, while the width of the emission slit was set to 5 nm.

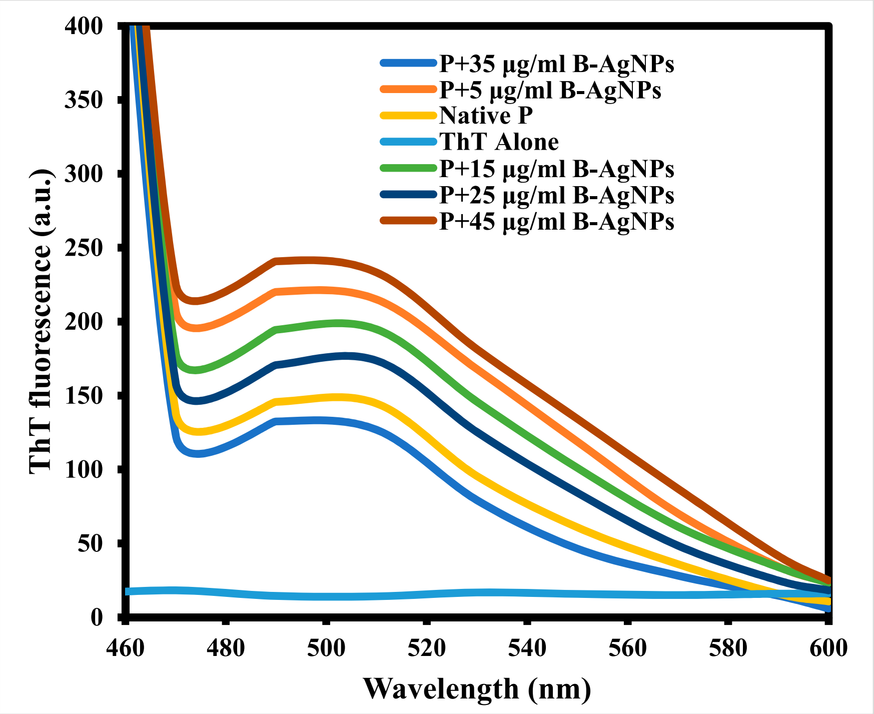

Figure S2. ThT fluorescence spectra.The fluorescence spectra of native HSA , HSA in the presence of increasing concentrations of B-AgNPs and ThT alone. HSA was used at a final concentration of 5 µM, while ThT was used at a final concentration of 20µM.

Reference

Ahmar Rauf, Mohd., Mohammad Oves, Fawad Ur Rehman, Abdur Rauf Khan, and Nazim Husain. 2019. "Bougainvillea Flower Extract Mediated Zinc Oxide's Nanomaterials for Antimicrobial and Anticancer Activity." *Biomedicine & Pharmacotherapy* 116:108983. doi: https://doi.org/10.1016/j.biopha.2019.108983.

Alam, Md. Tauqir, Mohd. Ahmar Rauf, Gufran Ahmed Siddiqui, Mohammad Owais, and Aabgeena Naeem. 2018a. "Green Synthesis of Silver Nanoparticles, Its Characterization, and Chaperone-like Activity in the Aggregation Inhibition of α-Chymotrypsinogen A." *International Journal of Biological Macromolecules* 120:2381–89. doi: https://doi.org/10.1016/j.ijbiomac.2018.09.006.

,

Oves, Mohammad, Mohd Ahmar Rauf, Afzal Hussain, Huda A. Qari, Aftab Aslam Parwaz Khan, Pir Muhammad, Md Tabish Rehman, Mohammad Fahad Alajmi, and Iqbal I. M. Ismail. 2019. "Antibacterial Silver Nanomaterial Synthesis From Mesoflavibacter Zeaxanthinifaciens and Targeting Biofilm Formation ." *Frontiers in Pharmacology* 10:801.
